# Polarization-resolved and polarization- multiplexed spike encoding properties in photonic neuron based on VCSEL-SA

**DOI:** 10.1038/s41598-018-34537-x

**Published:** 2018-10-31

**Authors:** Yahui Zhang, Shuiying Xiang, Xingxing Guo, Aijun Wen, Yue Hao

**Affiliations:** 1 State Key Laboratory of Integrated Service Networks, Xidian University, Xi’an, 710071 China; 2 State Key Discipline Laboratory of Wide Bandgap Semiconductor Technology, School of Microelectronics, Xidian University, Xi’an, 710071 China

## Abstract

The spike encoding properties of two polarization-resolved modes in vertical-cavity surface-emitting laser with an embedded saturable absorber (VCSEL-SA) are investigated numerically, based on the spin-flip model combined with the Yamada model. The results show that the external input optical pulse (EIOP) can be encoded into spikes in X-polarization (XP) mode, Y-polarization (YP) mode, or both XP and YP modes. Furthermore, the numerical bifurcation diagrams show that a lower (higher) strength of EIOP is beneficial for generating tonic (phasic) spikes; a small amplitude anisotropy contributes to wide (narrow) tonic spiking range in XP (YP) mode; a large current leads to low thresholds of EIOP strength for both XP and YP modes. However, the spike encoding properties are hardly affected by the phase anisotropy. The encoding rate is shown to be improved by increasing EIOP strength. Moreover, dual-channel polarization-multiplexed spike encoding can also be achieved in a single VCSEL-SA. To the best of our knowledge, such single channel polarization-resolved and dual-channel polarization-multiplexed spike encoding schemes have not yet been reported. Hence, this work is valuable for ultrafast photonic neuromorphic systems and brain-inspired information processing.

## Introduction

Vertical-cavity surface-emitting lasers (VCSELs) are popular candidates for many potential applications, including optical communication, optical signal processing, and optical computation, as they exhibit many advantages, such as low manufacturing cost, easy to integrate into two-dimensional arrays, and high energy efficiency^1,2^. In recent years, the nonlinear dynamics, polarization switching and polarization bistability properties of VCSELs have been intensively studied^3–14^. Interestingly, numerical and experimental investigations demonstrate that, by injecting external input optical pulse (EIOP), VCSELs can reproduce different behaviors exhibited by biological neurons including phasic spiking and tonic spiking, but on a much faster timescale^15–22^. Therefore, the VCSELs can be regarded as photonic neurons in the neuromorphic systems^18,20^.

Compared with conventional VCSELs, the VCSEL with an embedded saturable absorber (VCSEL-SA) can offer the excitability threshold for photonic neuron^16,21^. Based on a two-section rate equation model derived from the well-known Yamada model, some representative cortical spiking algorithms have been demonstrated numerically in small circuits consisting of excitable VCSELs-SA, including multistable circuit, synfire chain, and spatiotemporal pattern recognition circuit^16^. In addition, our previous numerical findings indicate that the spike codes can be stored successfully in the mutually coupled VCSELs-SA system^21^. However, the effects of polarization dynamics of VCSEL-SA on the spiking properties are not addressed in these works.

Moreover, the previous works mainly focused on single-channel spike encoding in the photonic neurons^15–23^. For example, single-channel all-optical digital-to-spike conversion was realized in the photonic neuron based on graphene excitable laser^23^. Hence, it is still open to explore whether dual-channel spike encoding can be achieved in a single VCSEL-SA taking advantage of the polarization dynamics.

In this paper, we focus on the numerically realization of dual-channel spike encoding in VCSEL-SA, and concentrate on the effects of polarization dynamics on the spiking encoding. By combining the well-known spin-flip model (SFM) and the Yamada model, the spike encoding properties in two polarization-resolved modes, including X-polarization (XP) and Y-polarization (YP) modes of the VCSEL-SA are investigated numerically in detail. The roles of EIOP strength, amplitude anisotropy, phase anisotropy and two pump currents are considered. Furthermore, we explore the dual-channel polarization-multiplexed spiking encoding in a single VCSEL-SA for the first time.

## Results

### Model

**Figure 1:**
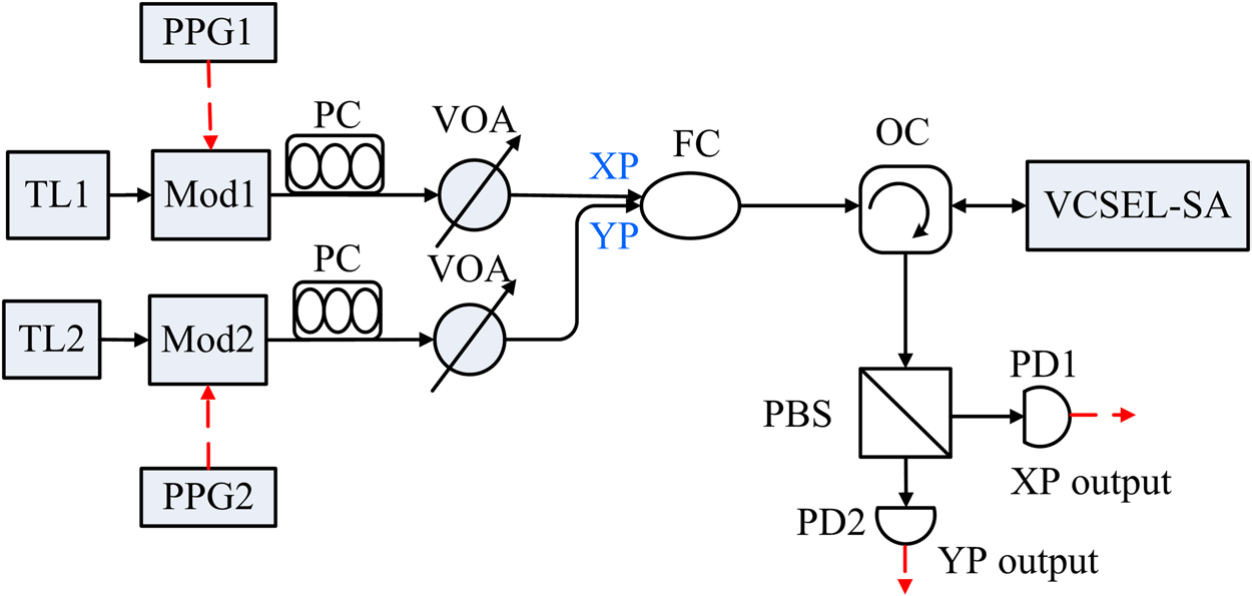
The schematic illustration of dual-channel spike encoding based on VCSEL-SA. TL1 and TL2: tunable laser; Mod1 and Mod2: modulator; PPG1 and PPG2: pulse signal generator; PC: polarization controller; VOA: variable optical attenuator; FC: fiber couple; OC: optical circulator; VCSEL-SA: vertical-cavity surface-emitting laser with an embedded saturable absorber; PBS: polarization beam splitter; PD1 and PD2: photodetector. The solid (dashed) lines correspond to optical (electrical) path.

The schematic diagram of dual-channel spike encoding based on VCSEL-SA is presented in Fig. 1. The output of TL1 (TL2) is considered as the optical input for XP (YP) mode. The pulses can be generated by PPG1 and PPG2. The polarization states of the two injected fields can be adjusted by PC. The output of FC is injected into the VCSEL-SA via OC. The VCSEL-SA device used in ref.^24^, which is composed of two InGaAs/AlGaAs quantum wells for the gain section, and one InGaAs/AlGaAs quantum well for the SA section, can be employed in our proposed scheme. The two polarization-resolved modes of the VCSEL-SA are then separated via a PBS for achieving two channel outputs. In a possible experiment, the strength of EIOP can be easily controlled by VOA.

### Formulation

The polarization dynamics of VCSEL can be described in the framework of the standard SFM^3,4^. The well-known Yamada model is successful for expressing a semiconductor laser with an embedded saturable absorber^16,21,25^. Our model is based on the combination of SFM and Yamada models^26,27^. We use two orthogonal linear components to replace two circularly polarized components as

1 $${F}_{x}=\frac{{F}_{+}+{F}_{-}}{\sqrt{2}},\,{F}_{y}=-\,i\frac{{F}_{+}-{F}_{-}}{\sqrt{2}}.$$

By including the polarized EIOPs, the rate equations can be written as follows:

2 $$\frac{d{F}_{x}}{dt}=\frac{1}{2}(1+i\alpha )[({D}_{1}+{D}_{2}-1){F}_{x}+i({d}_{1}+{d}_{2}){F}_{y}]-({\varepsilon }_{a}+i{\varepsilon }_{p}){F}_{x}+{k}_{injx}{F}_{injx}(t){e}^{i{\rm{\Delta }}{\omega }_{x}t}$$

3 $$\frac{d{F}_{y}}{dt}=\frac{1}{2}(1+i\alpha )[({D}_{1}+{D}_{2}-1){F}_{y}-i({d}_{1}+{d}_{2}){F}_{x}]+({\varepsilon }_{a}+i{\varepsilon }_{p}){F}_{y}+{k}_{injy}{F}_{injy}(t){e}^{i{\rm{\Delta }}{\omega }_{y}t}$$

4 $$\begin{array}{ccc}{D}_{1,2}^{\cdot } & = & {\gamma }_{1,2}[{\mu }_{1,2}-{D}_{1,2}-\frac{1}{2}{a}_{1,2}({D}_{1,2}+{d}_{1,2}){|{F}_{x}-i{F}_{y}|}^{2}\\  &  & -\,\frac{1}{2}{a}_{1,2}({D}_{1,2}-{d}_{1,2}){|{F}_{x}+i{F}_{y}|}^{2}+{c}_{12,21}{D}_{2,1}]\end{array}$$

5 $$\begin{array}{ccc}{d}_{1,2}^{\cdot } & = & -\,{\gamma }_{s1,2}{d}_{1,2}-{\gamma }_{1,2}[\frac{1}{2}{a}_{1,2}({D}_{1,2}+{d}_{1,2}){|{F}_{x}-i{F}_{y}|}^{2}\\  &  & -\,\frac{1}{2}{a}_{1,2}({D}_{1,2}-{d}_{1,2}){|{F}_{x}+i{F}_{y}|}^{2}-{c}_{12,21}{d}_{2,1}]\end{array}$$

where the subscripts 1 and 2 represent the pump and absorbing regions, respectively. *F*_*x*,*y*_ represent the slowly varying complex amplitudes of the two linear polarized components of the electric field. *D*_1,2_ represent the total carrier inversion between the conduction and valence bands related to the transparency carrier density. *d*_1,2_ represent the carrier inversions with opposite spin orientations. *ε*_*a*_ (*ε*_*p*_) is the amplitude (phase) anisotropy. The terms ${k}_{injx,y}{F}_{injx,y}(t){e}^{i{\rm{\Delta }}{\omega }_{x,y}t}$ in Eqs (2) and (3) denote the EIOPs, where *k*_*injx*,*y*_ are the input strength, *F*_*injx*,*y*_ characterize the optical pulse injection, and ${\rm{\Delta }}{\omega }_{x,y}$ is the angular frequency detuning between the injecting field and the VCSEL-SA. The terms *c*_12_*D*_2_ and *c*_21_*D*_1_ are the carrier diffusion. α is the linewidth enhancement factor, *μ*_1,2_ are the injection currents, *γ*_1,2_ are the total carrier decay rates, and *γ*_*s*1,2_ are the effective spin-flip rates. *a*_1,2_ are the differential gains. Here, we define ${\varepsilon }_{p}={\gamma }_{p}/\kappa$, where *γ*_*p*_ denotes the birefringence, and κ is the cavity decay rate. The equations are written in a dimensionless form so the time is measured in units of *κ*^−1^. The following parameters are used in simulation^26,27^: α = 3, *μ*_1_ = 2.1, *μ*_2_ = −6.1, *γ*_1_ = 1.09 × 10^−3^, *γ*_2_ = 1.13 × 10^−3^, *γ*_*s*1_ = *γ*_*s*2_ = 0.25, *ε*_*a*_ = 0, *γ*_*p*_ = 15 *ns*^−1^, *κ* = 390 *ns*^−1^, ${\rm{\Delta }}{\omega }_{x,y}=0\,rad/s$, *a*_1_ = 1, *a*_2_ = 8.7, *c*_12_ = 2.84 × 10^−2^, *c*_21_ = 1.91. With these parameters, the VCSEL-SA operates right below the lasing threshold *μ*_*th*_ = 2.6. Thus, without any external optical injection, no laser light is emitted^24^.

### Single-channel polarization-resolved spike encoding

In this section, we first consider polarization-resolved spike encoding in VCSEL-SA for two injection cases, (i) the EIOP is injected in XP mode, i.e., *k*_*injx*_ ≠ 0, *k*_*injy*_ = 0; (ii) the EIOP is injected in YP mode, i.e., *k*_*injx*_ = 0, *k*_*injy*_ ≠ 0. Then, the effects of EIOP strength, amplitude anisotropy, phase anisotropy and pump currents are studied. The distribution of time intervals between the encoding spikes is investigated.

**Figure 2:**
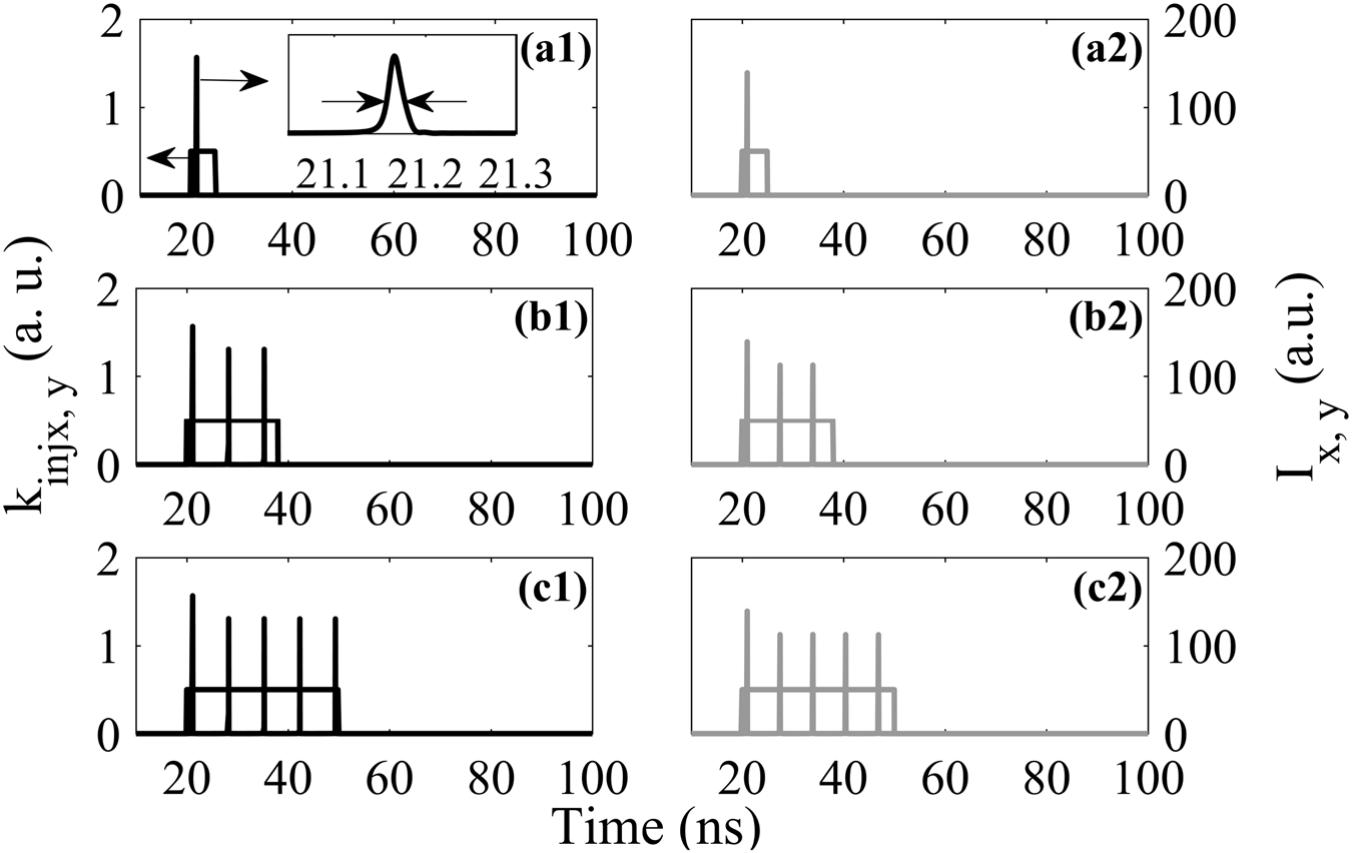
The polarization-resolved responses of VCSEL-SA subject to EIOP. (**a1**)–(**c1**) correspond to the case (i) with the during time 5 ns, 18 ns and 30 ns respectively. (**a2**)–(**c2**) correspond to the case (ii) with the during time 5 ns, 18 ns and 30 ns, respectively. The inset in (**a1**) indicates an enlargement of the generated spike.

Figure 2 indicates the responses of the polarization-resolved modes in VCSEL-SA subject to EIOPs. Here, three different temporal durations (denoted as Δτ) are considered for both injection cases. The shape of the EIOP is rectangular with *k*_*injx*,*y*_ = 0.5. The output intensities are denoted as ${I}_{x}={|{F}_{x}|}^{2}$ and ${I}_{y}={|{F}_{y}|}^{2}$, respectively. It can be seen from Fig. 2(a1) that, for case (i), when Δτ = 5 ns, one spike is responded in XP mode. Note that, the solitary VCSEL-SA operates right below its lasing threshold, and the EIOP brings the laser above the threshold and triggers the firing of spikes. The full width at half maximum (FWHM) of the generated spike is denoted as *τ*_*FWHM*_, and is *τ*_*FWHM*_ = 17 *ps* as shown in the inset of Fig. 2(a1). For Δτ = 18 ps (Δτ = 30 ps), it can be seen from Fig. 2(b1),(c1), three (five) spikes are obtained in the XP mode. Furthermore, the value of *τ*_*FWHM*_ is the same as that in Fig. 2(a1). As can be seen in Fig. 2(a2)–(c2), for case (ii), with different Δτ of EIOP, the EIOP is encoded into one, three, and five spikes in YP mode, respectively, which are similar to the responses in XP mode. The *τ*_*FWHM*_ is also 17 ps. That is to say, the EIOP can be successfully encoded into spikes in two polarization-resolved modes, respectively. Besides, a large Δτ leads to more spikes in the polarization-resolved modes for both injection cases, while the FWHM of the generated spikes is hardly affected by Δτ.

**Figure 3:**
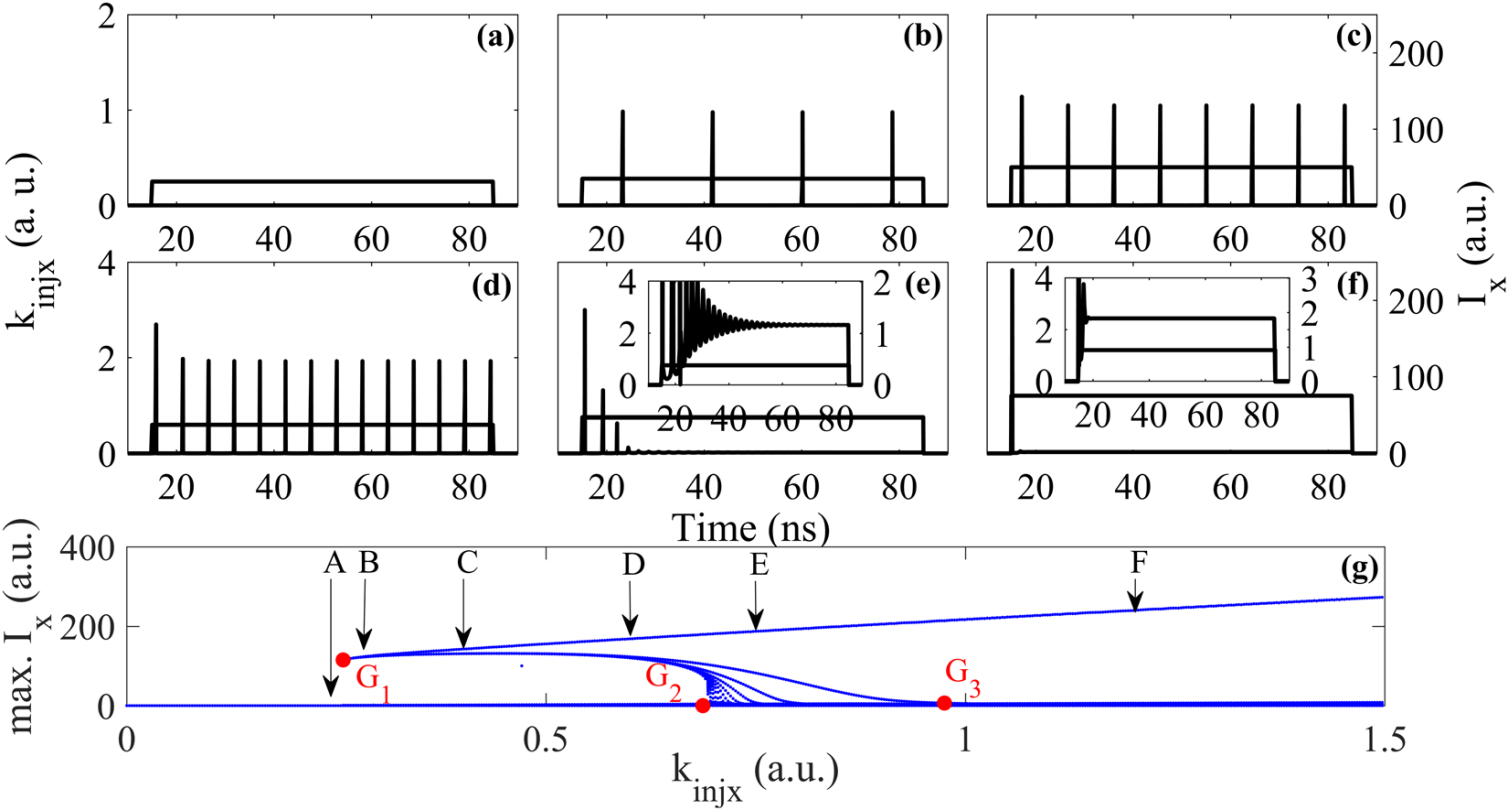
The responses of VCSEL-SA to EIOP subject XP mode (**a**)–(**f**) and the numerical bifurcation diagram of XP mode as a function of *k*_*injx*_ (**g**). (**a**)–(**f**) with *k*_*injx*_ = 0.25, 0.28, 0.4, 0.6, 0.75, 1.2. The enlargements of the generated spikes of (**e**),(**f**) are plotted in the insets.

Next, the effect of EIOP strength on the spiking encoding in the polarization-resolved modes is studied. As the response properties in YP mode are similar to those in XP mode, we only present the spike encoding properties in XP mode for simplicity. The responses in XP mode of VCSEL-SA for different EIOP strengths are presented in Fig. 3(a)–(f). Here, we consider Δτ = 70 ns. It can be seen from Fig. 3(a), when *k*_*injx*_ = 0.25, no spike is generated, indicating that the strength is below the excitability threshold. From Fig. 3(b)–(d), we can see that, when *k*_*injx*_ = 0.28, 0.4 and 0.6, the responses of VCSEL-SA during the EIOP are multiple periodic spikes, and the corresponding *τ*_*FWHM*_ is 19 ps, 18 ps and 17 ps, respectively. Interestingly, such multiple periodic spikes are similar to the tonic spiking obtained experimentally and numerically based on the SFM in conventional VCSEL^15,17^. Moreover, the intervals between two consecutive spikes decrease with the increase of *k*_*injx*_. Also, the intensity of the first spike is increased with the increasing *k*_*injx*_. The response for *k*_*injx*_ = 0.75 is presented in Fig. 3(e), the output consists of one large intensity spike (with *τ*_*FWHM*_ = 16 ps) and some damped spikes. Eventually, the intensity is about 1 in the remaining duration of the EIOP, as shown in the inset. When the *k*_*injx*_ is further increased, only one large intensity spike (with *τ*_*FWHM*_ = 15 ps) is generated in XP mode upon the arrival of EIOP as shown in Fig. 3(f) for *k*_*injx*_ = 1.2, which is similar to the phasic spiking reported in conventional VCSEL^15,17^. It also can be seen from the inset, the intensity is about 2 in the remaining duration of the EIOP. Note that, the stable states shown in Fig. 3(e) and (f), originate from the injection-locking effect. That is to say, the large intensity spike is the result of a transitory effect produced by switching transitions between the non-lasing state and the injection locking state^15^.

It has already been shown that, the bifurcation diagram is a useful tool to characterize different neuron-like dynamics^20,22^. The phasic spiking and tonic spiking have been identified in conventional VCSEL^20^. We also present the bifurcation diagram, i.e., the maximum of *I*_*x*_ as a function of *k*_*injx*_, in Fig. 3(g). It can be found that, the excitability threshold is *k*_*injx*_ = 0.26, corresponding to the red dot G_1_. When *k*_*injx*_ < 0.26, no spike is generated, corresponding to the response shown in Fig. 3(a) for *k*_*injx*_ = 0.25 shown by arrow A. When 0.26 < *k*_*injx*_ < 0.69 (red dot G_2_), for the pointed arrows B, C and D corresponding to the *k*_*injx*_ used in Fig. 3(b)–(d), tonic spiking is obtained. When 0.69 < *k*_*injx*_ < 0.98 (red dot G_3_), a large intensity spike followed by damped spikes is observed, a representative case is shown in Fig. 3(e), shown by the arrow E corresponding to the *k*_*injx*_ = 0.75. When *k*_*injx*_ < 0.98, phasic spiking is obtained, which can be seen clearly from Fig. 3(f) corresponding to arrow F.

**Figure 4:**
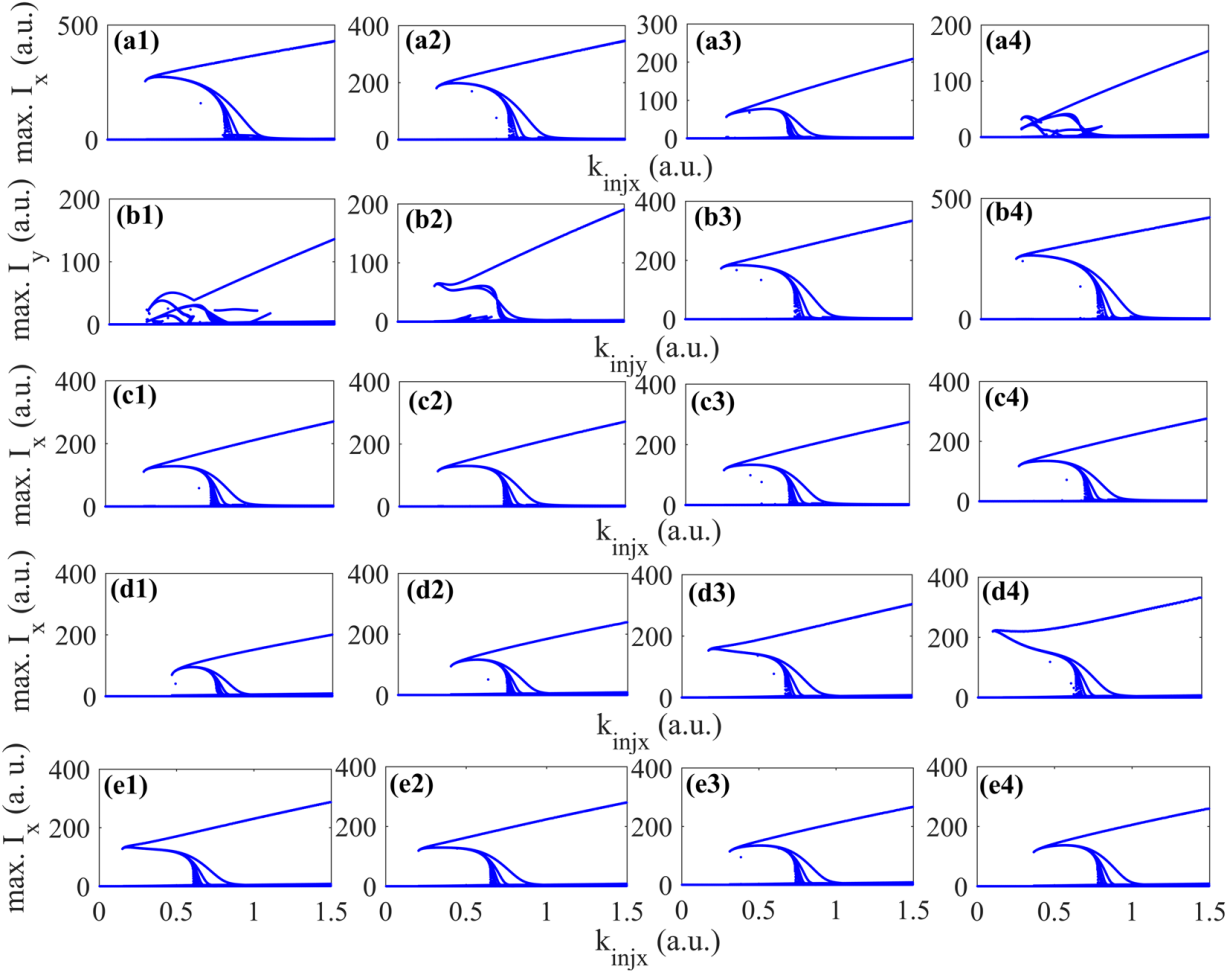
Numberical bifurcation diagrams as a function of *k*_*injx*_ under different cases of *ε*_*a*_ in XP mode (**a1**)–(**a4**), *ε*_*a*_ in YP mode (**b1**)–(**b4**), *ε*_*p*_ in XP mode (**c1**)–(**c4**), *μ*_1_ in XP mode (**d1**)–(**d4**) and *μ*_2_ in XP mode (**e1**)–(**e4**). (**a1**)–(**a4**), (**b1**)–(**b4**) corresponding to *ε*_*a*_ = −0.1, −0.05, 0.05 and 0.1, (**c1**)–(**c4**) corresponding to *γ*_*p*_ = 9, 12, 18 and 21 ns^−1^, (**d1**)–(**d4**) corresponding to *μ*_1_ = 1.9, 2.0, 2.2 and 2.3, (**e1**)–(**e4**) corresponding to *μ*_2_ = −5.7, −5.9, −6.3 and −6.5.

Next, we use the bifurcation diagram to further analyze the effect of different parameters on spiking coding characteristics. Figure 4 presents the bifurcation diagram for the response as a function of *k*_*injx*,*y*_ under different cases of amplitude anisotropy, phase anisotropy and injection currents. The other parameters are identical to those in Fig. 3(g). For injection case (i), bifurcation diagrams for XP mode as a function of *k*_*injx*_ under different amplitude anisotropy *ε*_*a*_ are presented in Fig. 4(a1)–(a4). It can be seen that, the excitability threshold is approximately *k*_*injx*_ = 0.26 for all different cases of *ε*_*a*_. Besides, the range of *k*_*injx*_ corresponding to tonic spiking is 0.26 < *k*_*injx*_ < 0.74, for *ε*_*a*_ = −0.1, 0.26 < *k*_*injx*_< 0.72 for *ε*_*a*_ = −0.05, 0.26 < *k*_*injx*_ < 0.66 for *ε*_*a*_ = 0.05 and 0.27 < *k*_*injx*_ < 0.62 for *ε*_*a*_ = 0.1. In addition, the range of *k*_*injx*_ corresponding to phasic spiking is *k*_*injx*_ > 1.06 for *ε*_*a*_ = −0.1, *k*_*injx*_ > 1.05 for *ε*_*a*_ = −0.05, *k*_*injx*_ > 0.94 for *ε*_*a*_ = −0.05 and *k*_*injx*_ > 0.84 for *ε*_*a*_ = 0.1. Namely, for a larger *ε*_*a*_, the range of *k*_*injx*_ corresponding to tonic spiking is narrower, and the minimum *k*_*injx*_ corresponding to the onset of phasic spiking moves to a lower value. Correspondingly, for injection case (ii), the bifurcation diagrams for YP mode as a function of *k*_*injy*_ under different *ε*_*a*_ are presented in Fig. 4(b1)–(b4). It can be seen that, when *ε*_*a*_ = −0.1, −0.05, 0.05 and 0.1, the ranges of *k*_*injy*_ corresponding to the tonic spiking are 0.24 < *k*_*injx*_ < 0.66, 0.24 < *k*_*injx*_ < 0.67, 0.24 < *k*_*injx*_ < 0.72, and 0.23 < *k*_*injx*_ < 0.77, respectively. Additionally, the minimum value of *k*_*injy*_ corresponding to the onset of phasic spiking is 0.81, 0.89, 1.06 and 1.14, respectively. That is to say, the excitability threshold is also approximately the same for different *ε*_*a*_ in YP mode. Besides, the range of *k*_*injy*_ corresponding to tonic spiking is broadened for a larger *ε*_*a*_, while the minimum *k*_*injy*_ corresponding to the onset of phasic spiking moves to higher value, which contrasts with the injection case (i). The numerical bifurcation diagrams for different *ε*_*p*_ are shown in Fig. 4(c1)–(c4) for injection case (i). It can be seen that, the four bifurcation diagrams are similar, and are also identical to Fig. 3(g). That is to say, the spike encoding property is hardly affected by *ε*_*p*_. The bifurcation diagrams for injection case (i) under different *μ*_1_ are presented in Fig. 4(d1)–(d4). It can be seen that, a larger *μ*_1_ leads to a lower excitability threshold. More precisely, the value of excitability threshold is 0.44, 0.35, 0.17 and 0.11 for *μ*_1_ = 1.9, 2.0, 2.2 and 2.3, respectively. Besides, a larger *μ*_1_ contributes to a wider range of *k*_*injx*_ corresponding to tonic spiking. In addition, the *k*_*injx*_ corresponding to the end of tonic spiking is 0.73, 0.71, 0.67 and 0.65 for *μ*_1_ = 1.9, 2.0, 2.2 and 2.3, respectively. Note that, the minimum values of *k*_*injx*_ corresponding to the onset of phasic spiking are almost similar for different cases of *μ*_1_. The bifurcation diagrams for different *μ*_2_ are presented in Fig. 4(e1)–(e4) for injection case (i). It can be seen that, the value of excitability threshold is 0.16, 0.21, 0.32 and 0.38 for *μ*_2_ = −5.7, −5.9, −6.3 and −6.5, respectively, indicating that a larger *μ*_2_ also leads to a lower excitability threshold. Besides, the ranges of *k*_*injx*_ corresponding to tonic spiking are almost similar for different cases of *μ*_2_. The *k*_*injx*_ corresponding to the end of tonic spiking is 0.60, 0.65, 0.74 and 0.77 for *μ*_2_ = −5.7, −5.9, −6.3 and −6.5, respectively. In addition, the minimum *k*_*injx*_ corresponding to the onset of phasic spiking is 0.89, 0.94, 1.05 and 1.06 for *μ*_2_ = −5.7, −5.9, −6.3 and −6.5, respectively. The bifurcation diagrams of YP mode for injection case (ii) are similar to those of XP mode for different cases of *ε*_*p*_, *μ*_1_, and *μ*_2_, and are not shown here.

**Figure 5:**
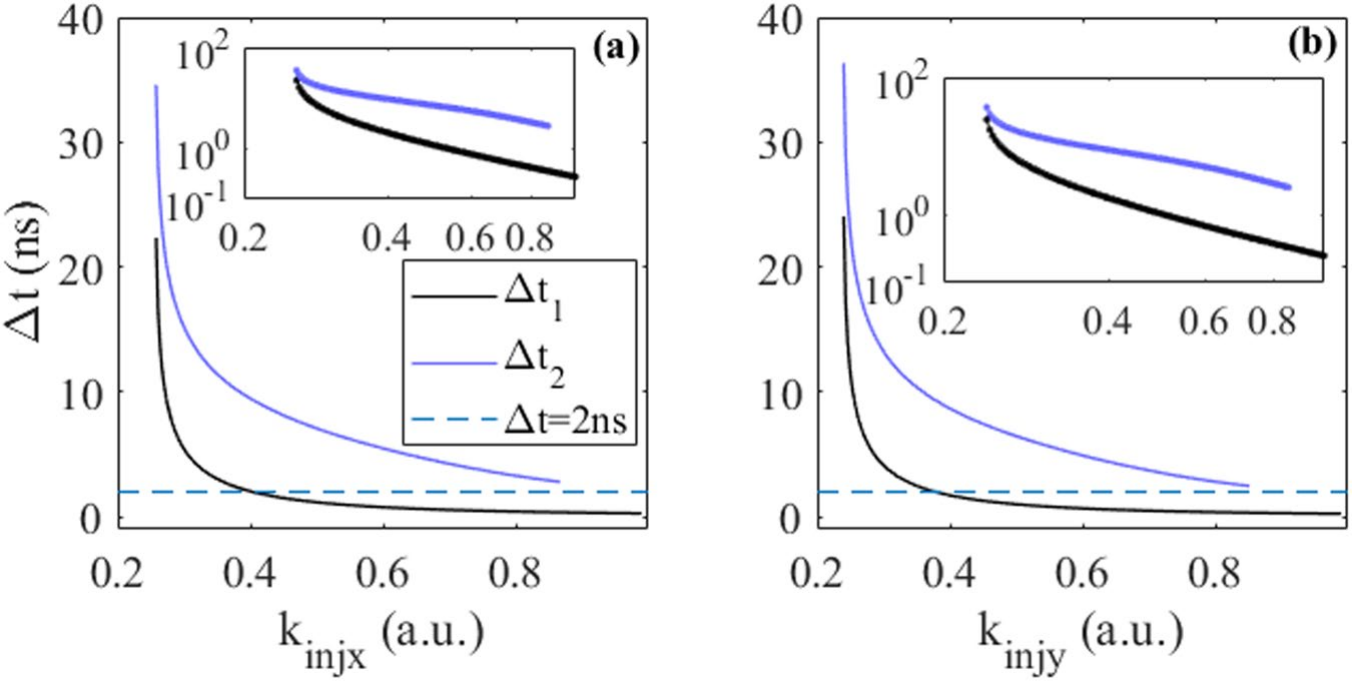
Δ*t*_1_ (black solid line) and Δ*t*_2_ (purple solid line) as functions of *k*_*injx*_ under XP mode (**a**) and *k*_*injy*_ under YP mode (**b**). The dashed blue line represent Δt = 2 ns. The insets are the corresponding log-log plots.

The time interval between the onset of EIOP and the first output spike (denoted as Δ*t*_1_^21^) and the time interval between two consecutive output spikes (denoted as Δ*t*_2_^21^) as functions of *k*_*injx*_ (*k*_*injy*_) for both injection cases are presented in Fig. 5. Here, we also consider Δτ = 70 ns. On the one hand, for injection case (i), it can be seen from Fig. 5(a) that, with the increase of *k*_*injx*_, the values of Δ*t*_1_ decrease sharply and then converge, while the values of Δ*t*_2_ decrease sharply and then disappear when *k*_*injx*_ > 0.88. The values of Δ*t*_1_ are close to 0.27 ns when *k*_*injx*_ approach 1. For clarity, the values of Δ*t*_1_ and Δ*t*_2_ versus *k*_*injx*_ are also presented in log-log plots as shown in the inset. Moreover, we find that, the value of Δ*t*_2_ is always greater than 2 ns (blue dashed line). Hence, the Δτ can be fixed at 2 ns to ensure a single spike output for a given EIOP. Note, the *k*_*injx*_ should be large enough and above the excitability threshold, that is, *k*_*injx*_ = 0.41 for Δτ = 2 ns. On the other hand, it can be seen in Fig. 5(b) that, similar results are found for injection case (ii). In this way, temporal spike encoding based on the spike latency, i.e., Δ*t*_1_, can be achieved in the VCSEL-SA^28,29^.

**Figure 6:**
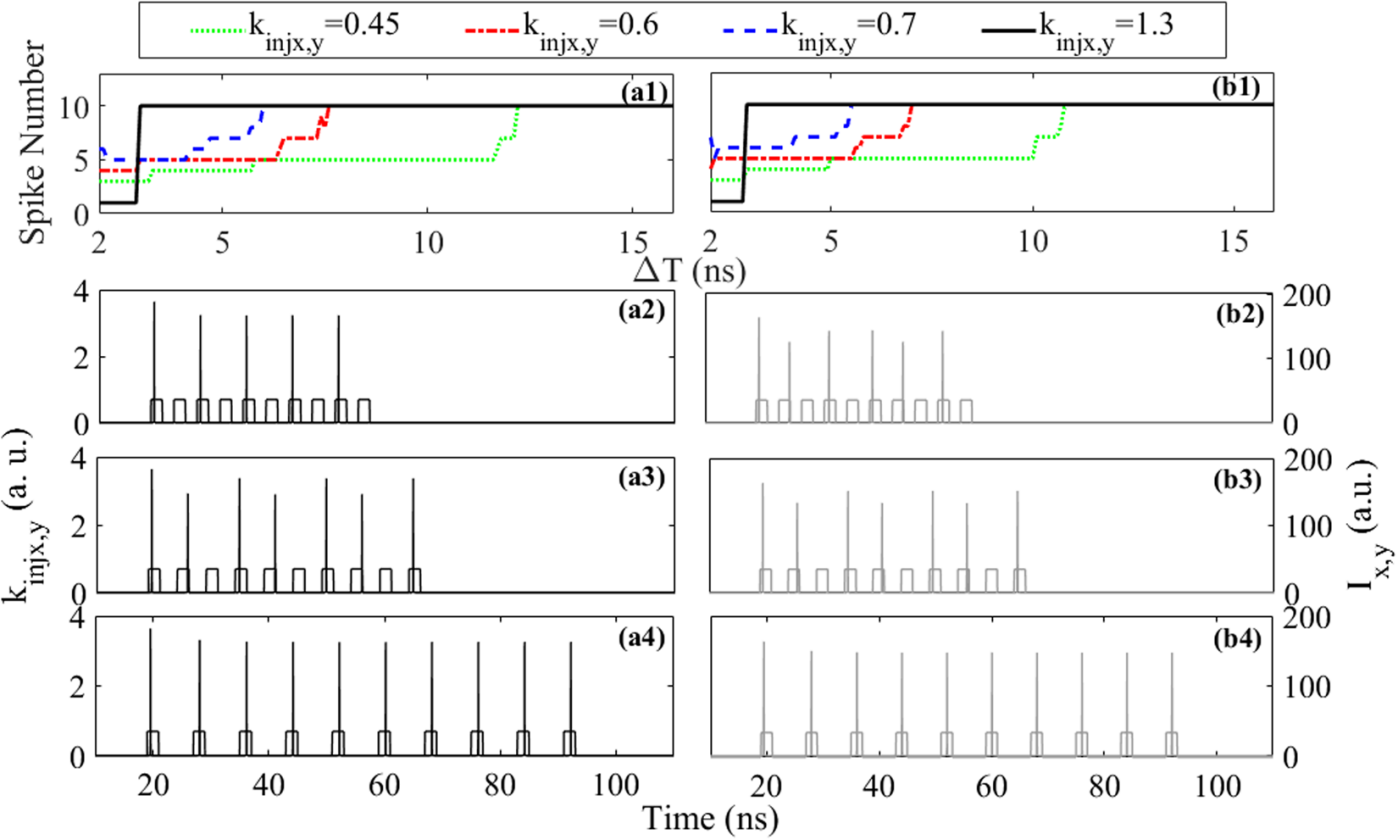
The spike number as functions of bit period ΔT (**a1**) for XP mode and (**b1**) YP mode. The time series of responses (**a2**)–(**a4**) in the XP mode and (**b2**)–(**b4**) YP mode, with ΔT = 4 ns in (**a2**) and (**b2**), ΔT = 5 ns in (**a3**) and (**b3**), and ΔT = 8 ns in (**a4**) and (**b4**).

Next, we further discuss the encoding rate, which is closely related to the refractory period of a photonic neuron^23^. For simplicity, the return-to-zero (RZ) sequence, whose period is denoted as ΔT, is regarded as EIOP. Here, ten bits of 1 are considered, and the pulse width in one bit is fixed at 2 ns. In this way, the successful one-bit-to-one-spike encoding is identified by ten spikes in the response. For convenience, a successful spike is characterized by max. *I*_*x*,*y*_ ≥ 20 in the given bit duration. On the one hand, for injection case (i), the output spike numbers as functions of ΔT for four different *k*_*injx*_ are presented in Fig. 6(a1). It can be seen that, the spike number increases step-wise with the increase of ΔT and finally reaches ten at ΔT = 12.2 ns for *k*_*injx*_ = 0.45, at ΔT = 7.6 ns for *k*_*injx*_ = 0.6, at ΔT = 6 ns for *k*_*injx*_ = 0.7 and at ΔT = 3 ns for *k*_*injx*_ = 1.3, respectively. That is to say, the ten bits can be encoded correctly in the XP mode only when ΔT is sufficiently large, which can be attributed to the refractory period^28,29^. Besides, for a larger *k*_*injx*_, the output spike number reaches ten at a smaller ΔT. Thus, the encoding rate, which is equal to 1/ΔT, can be improved by increasing *k*_*injx*_. Usually, the encoding rate can be further improved by decreasing the volumes of the cavities and material recombination times^24^. On the other hand, for injection case (ii), the output spike numbers as functions of ΔT for four different *k*_*injy*_ are presented in Fig. 6(b1). We can find that, the minimum ΔT corresponding to ten spikes is ΔT = 10.8 ns for *k*_*injy*_ = 0.45, ΔT = 7 ns for *k*_*injy*_ = 0.6, ΔT = 5.5 ns for *k*_*injy*_ = 0.7 and ΔT = 2.9 ns for *k*_*injy*_ = 1.3, respectively. Namely, the ten bits can also be encoded correctly in YP mode for sufficiently large ΔT, and higher encoding rate can be achieved for larger *k*_*injy*_. In order to intuitively present the encoding process, the time series of responses in the XP mode for three representative ΔT are presented in Fig. 6(a2)–(a4) with *k*_*injx*_ = 0.7. It can be clearly seen that, ten bits are encoded into five spikes for ΔT = 4 ns, and seven spikes for ΔT = 5 ns. Namely, the one-bit-to-one-spike encoding fails due to the refractory period. For ΔT = 8 ns, ten bits are successfully encoded into ten spikes. Correspondingly, the time series of responses in the YP mode for three representative cases of ΔT are presented in Fig. 6(b2)–(b4), and similar results are obtained.

### Dual-channel polarization-multiplexed spike encoding

**Figure 7:**
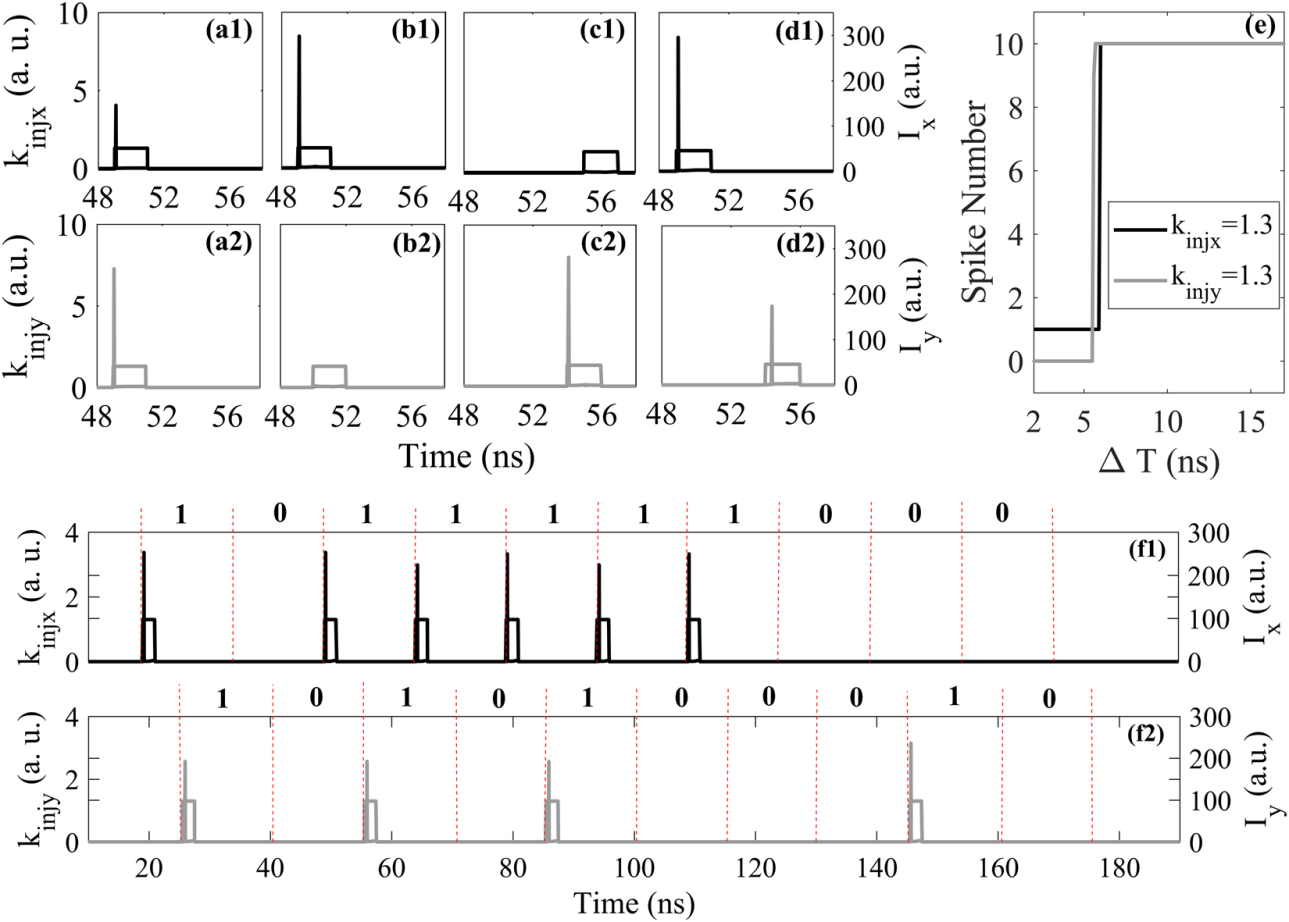
The response outputs of VCSEL-SA subject to two EIOPs for four representative cases. (**a**) the time windows of EIOPs are completely overlapped, (**b**) the EIOP injected into XP arrives earlier,(**c**) the EIOP injected into YP arrives earlier, (**d**) two EIOPs are well separated. (**e**) Spike numbers as functions of ΔT for *k*_*injx*_ = 1.3 and *k*_*injy*_ = 1.3. The response outputs of VCSEL-SA subject to pseudo-random bit sequences in XP (**f1**) and YP (**f2**) mode for ΔT = 15 ns.

Next, we explore the polarization-multiplexed spike encoding in a single VCSEL-SA. That is to say, two different EIOPs are injected in both XP and YP modes, respectively, i.e., *k*_*injx*_ ≠ 0, *k*_*injy*_ ≠ 0. Through extensive calculation, we find that when *k*_*injx*_ and *k*_*injy*_ are relative small, the polarization mode competition and multiple polarization switching will occur and seriously affect the tonic spiking dynamics, leading to very complex spike encoding properties. Hence, for simplicity, we only consider large values of *k*_*injx*_ and *k*_*injy*_ corresponding to phasic spiking dynamics. The responses in both XP and YP modes for four representative cases are shown in Fig. 7. Here, we consider Δτ = 70 ns, *k*_*injx*_ = 1.3 and *k*_*injy*_ = 1.3. It can be seen that, when the time windows of EIOPs are completely overlapped as shown in Fig. 7(a1) and (a2), the phasic spike is responded shortly after the arrival of EIOPs in both XP and YP modes. In this case, the EIOPs arrive simultaneously, hence both XP and YP mode share the carriers in VCSEL-SA. However, when the time windows of two EIOPs are partially overlapped, as can be seen in Fig. 7(b1),(b2),(c1) and (c2), the phasic spike can only be achieved in the XP (YP) mode when the EIOP received by the XP (YP) mode is arrived earlier. Note that, the spike triggered by the earlier arrival leads to carrier depletion via polarization mode competition^7,10^. The threshold cannot be reached to trigger another spike in the other mode. Interestingly, such behavior is quite similar to a biological behavior of an inhibitory interneuron that hinders the firing of others^30^. As presented in Fig. 7(d1) and (d2), the phasic spikes can also be generated successfully in both XP and YP modes when the two EIOPs are well separated. We have also discussed the encoding rate for the polarization-multiplexed spike encoding. To ensure successful dual-channel polarization-multiplexed spike encoding in a single VCSEL-SA, two sequences with half-period difference are injected into both XP and YP modes. The output spike numbers in XP and YP modes as functions of ΔT are further presented in Fig. 7(e). Here, a representative case of *k*_*injx*_ = 1.3, *k*_*injy*_ = 1.3 is considered. It can be seen that, when ΔT > 6 ns (ΔT > 5.7 ns), ten spikes can be generated in XP (YP) mode. Hence dual-channel polarization-multiplexed spike encoding can be achieved in a single VCSEL-SA when ΔT > 6 ns. Besides, to obtain more general results, we also consider the pseudo-random bit sequence as the EIOP. Here, the response outputs for only one representative ΔT is presented. It can be seen from Fig. 7(f1) and (f2) that, ten bits of pseudo-random sequence are encoded successfully in both XP and YP mode for *k*_*injx*_ = 1.3 and *k*_*injy*_ = 1.3. Note that, the polarization-multiplexed spike encoding scheme guarantees dual-channel parallel information processing in a single VCSEL-SA, which leads to reduced system cost. Hence, it is valuable for the implementation of parallel photonic information processing and photonic neuromorphic systems. Moreover, the polarization-multiplexed spike encoding may also be interesting for two channel vector coding^31^.

### Robustness to noise

**Figure 8:**
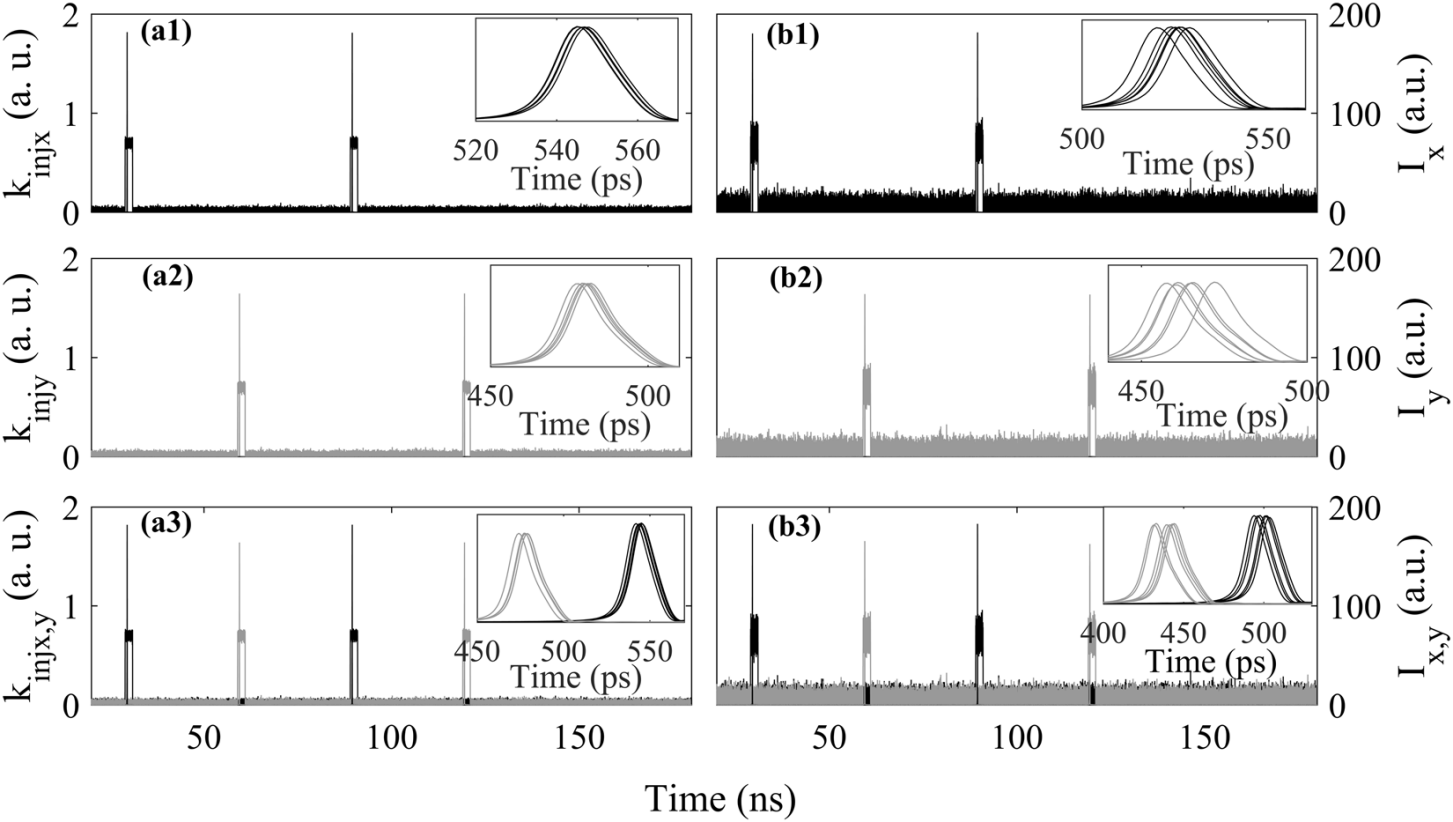
Numerical simulation of RZ bit sequence encoding in XP mode ((**a1**) and (**b1**)), YP mode ((**a2**) and (**b2**)), and both in XP and YP modes ((**a3**) and (**b3**)), when the RZ bit sequence has an SNR = 30 dB ((**a1**)–(**a3**)), SNR = 20 dB ((**b1**)–(**b3**)). Insets: eye diagrams corresponding to (**a1**)–(**b3**), respectively.

Without loss of generality, we also consider the effect of signal noise on the spike encoding properties. Here, white Gaussian noise is added to the RZ sequences^23^. The encoding outputs for different signal-to-noise ratios (SNR) are presented in Fig. 8 for three injection cases mentioned above. To ensure the successful spike encoding for all the injection cases, we select ΔT = 60 ns, *k*_*injx*_ = 0.7 and *k*_*injy*_ = 0.7. It can be seen from Fig. 8(a1),(a2),(b1) and (b2) that, for both SNR = 30 dB and SNR = 20 dB, two spikes can be generated in XP or YP modes in VCSEL-SA. Moreover, for both cases of SNR, two spikes can be achieved in a single VCSEL-SA by polarization-multiplexed spike encoding. Hence, the polarization-resolved and polarization-multiplexed spike encoding schemes in VCSEL-SA are robust to noisy RZ sequences, which is similar to the finding obtained in a graphene excitable laser^23^. The white Gaussian noises induced timing jitters of output spikes are presented in the insets in Fig. 8(a1)–(a3) by eye diagrams^23^ of six spikes. It can be seen that, the timing jitter^23^ is about 2 ps (9 ps) for SNR = 30 dB (20 dB). Compared to the values of Δ*t*_1_ and Δ*t*_2_ shown in Fig. 5, the timing jitters are small and negligible. Hence, in temporal coding process, the timing jitter can be neglected by properly selecting the EIOP strength.

## Discussion

In the present work, we derive the theoretical model to account for the polarization dynamics, saturable absorber, and EIOP in VCSEL-SA, by combing the well-known SFM and Yamada models. The single-channel spike encoding in the polarization-resolved modes and the dual-channel polarization-multiplexed spike encoding in a single VCSEL-SA are investigated numerically. The results show that the EIOPs can be encoded into spikes in XP mode, YP mode, or both XP and YP mode in VCSEL-SA. Besides, the generated spikes are similar to the tonic spiking and phasic spiking observed in traditional VCSEL. In addition, the numerical bifurcation analyses indicate that a small value of EIOP strength is beneficial for generating tonic spiking; a large value of EIOP strength is beneficial for generating phasic spiking; a small amplitude anisotropy contributes to a wide (narrow) range of tonic spiking in XP (YP) mode; large pump currents lead to low excitability threshold. While the spike encoding properties are hardly affected by the phase anisotropy. In the context of single-channel spike encoding, the encoding rate can be improved by increasing input strength. Furthermore, the polarization-multiplexed spike encoding is achieved in a single VCSEL-SA under proper condition. At last, we find that the polarization-resolved spike encoding as well as the polarization-multiplexed spike encoding are robust to noisy RZ sequences. To the best of our knowledge, such polarization-resolved and polarization-multiplexed spike encoding have not yet been reported, and are interesting and valuable for the ultrafast photonic neuromorphic systems and brain-inspired photonic information processing.

## Methods

The Eqs (2)–(5) for VCSEL-SA are simulated in the MATLAB platform. They have been integrated by using a fourth-order Runge-Kutta algorithm. Specifically, each time series has been obtained by running the program with a fixed time step of 1 ps.
